# NKG2D-CAR-transduced natural killer cells efficiently target multiple myeloma

**DOI:** 10.1038/s41408-021-00537-w

**Published:** 2021-08-14

**Authors:** Alejandra Leivas, Antonio Valeri, Laura Córdoba, Almudena García-Ortiz, Alejandra Ortiz, Laura Sánchez-Vega, Osvaldo Graña-Castro, Lucía Fernández, Gonzalo Carreño-Tarragona, Manuel Pérez, Diego Megías, María Liz Paciello, Jose Sánchez-Pina, Antonio Pérez-Martínez, Dean A. Lee, Daniel J. Powell, Paula Río, Joaquín Martínez-López

**Affiliations:** 1 H12O-CNIO Haematological Malignancies Clinical Research Unit, Spanish National Cancer Research Centre, Madrid, Spain; 2 Department of Hematology, Hospital Universitario 12 de Octubre—Universidad Complutense, Instituto de Investigación Sanitaria Hospital 12 de Octubre (imas12), Madrid, Spain; 3 Bioinformatics Group, Spanish National Cancer Research Centre, Madrid, Spain; 4 Confocal Microscopy Unit, Spanish National Cancer Research Centre, Madrid, Spain; 5 Department of Pediatrics, Hospital Universitario La Paz, Madrid, Spain; 6 Cellular Therapy and Cancer Immunology Program, The Research Institute at Nationwide Children’s Hospital, Columbus, OH USA; 7 Department of Pathology and Laboratory Medicine, University of Pennsylvania, Philadelphia, PA USA; 8 Division of Hematopoietic Innovative Therapies, Centro de Investigaciones Energéticas Medioambientales y Tecnológicas and Centro de Investigación Biomédica en Red de Enfermedades Raras (CIEMAT/CIBERER), Madrid, 28040 Spain; 9 Advanced Therapies Unit, Instituto de Investigación Sanitaria Fundación Jiménez Díaz (IIS-FJD, UAM), Madrid, 28040 Spain

**Keywords:** Translational research, Cancer immunotherapy, Myeloma, Immunotherapy

## Abstract

CAR-T-cell therapy against MM currently shows promising results, but usually with serious toxicities. CAR-NK cells may exert less toxicity when redirected against resistant myeloma cells. CARs can be designed through the use of receptors, such as NKG2D, which recognizes a wide range of ligands to provide broad target specificity. Here, we test this approach by analyzing the antitumor activity of activated and expanded NK cells (NKAE) and CD45RA^−^ T cells from MM patients that were engineered to express an NKG2D-based CAR. NKAE cells were cultured with irradiated Clone9.mbIL21 cells. Then, cells were transduced with an NKG2D-4-1BB-CD3z-CAR. CAR-NKAE cells exhibited no evidence of genetic abnormalities. Although memory T cells were more stably transduced, CAR-NKAE cells exhibited greater in vitro cytotoxicity against MM cells, while showing minimal activity against healthy cells. In vivo, CAR-NKAE cells mediated highly efficient abrogation of MM growth, and 25% of the treated mice remained disease free. Overall, these results demonstrate that it is feasible to modify autologous NKAE cells from MM patients to safely express a NKG2D-CAR. Additionally, autologous CAR-NKAE cells display enhanced antimyeloma activity demonstrating that they could be an effective strategy against MM supporting the development of NKG2D-CAR-NK-cell therapy for MM.

## Introduction

New-generation drugs have improved the quality of life and survival among multiple myeloma (MM) patients [1], but curative therapy remains challenging. Most patients suffer multiple relapses and develop refractory disease [2]. MM patients exhibit immune system dysregulation. MM patients have significantly reduced NK-cell counts during advanced stages of disease [3]. More so, NK cells in MM patients display limited cytotoxic activity [4].

Several groups have developed methods to modify lymphocytes for improved antitumor efficacy [5–7], and these investigations confirm that harnessing the immune system is a promising anticancer therapeutic strategy. In spite of encouraging preclinical results, these have not been directly translated to improved clinical responses and outcomes in MM [8].

Chimeric antigen receptor (CAR) cell products can further augment immune potential [9]. CAR cells are usually restricted to recognize a single tumor antigen on a limited subset of tumors, but they can also be targeted using nonantibody approaches, through the extracellular binding domain of natural receptors [10]. One promising candidate receptor for CAR production is NKG2D a C-type lectin-like innate activating receptor expressed on most human NK cells, and by activated CD8^+^ T cells, NKT cells, and some T-cell subsets (CD4^+^ and γδ^+^) [11]. NKG2D recognizes and kills infected cells and cancer cells by targeting up to eight stress-induced ligands (NKG2DL), namely MICA, MICB, and ULBP [1–6] that are overexpressed on viral infected, DNA-damaged or transformed cells, and not usually expressed in healthy tissues [12, 13]. Over 70% of human cancers upregulate NKG2D ligand expression [14–16]. Previous studies have shown that NK cells are effective against tumor cells expressing NKG2DL in their membrane [17].

CAR-T cells are most commonly used effector cell type; however, therapy with CAR-T cells can be associated with life-threatening toxic effects, such as cytokine release syndrome (CRS) and neurotoxicity [18, 19]. Additionally, tumor cells may develop immune escape strategies such as antigen loss, preventing CAR-T-cell recognition. In contrast, CAR-transduced NK cells are short-lived cells with more transient toxicities [20]. Moreover, NK-cell cytotoxicity can be triggered in a CAR-independent manner via stimulatory and inhibitory receptors, increasing the antitumor activity [21].

Our group has delivered large numbers of activated and expanded autologous NK cells (termed NKAE), which showed good clinical results without toxicity [4], establishing a possible platform for autologous CAR-NKAE development. Here we examined the merging of NK-CAR and NKAE therapies, by generating CAR-transduced NKAE-cell products, which may be highly beneficial for MM treatment. We also compared the in vitro efficacy between CAR-NKAE-cell products versus memory CAR-T cells [22].

## Materials and methods

### Samples and cell lines

Investigations were performed using fresh peripheral blood (PB) samples from MM patients (Supplemental Table S1) and healthy donors, and bone marrow (BM) samples from patients with newly diagnosed or relapsed MM.

Clone9.mbIL21 cells were used as previously described [6]. MM cell lines and healthy cell lines were cultured as described in the Supplementary Methods (Supplementary Material File).

### CAR-expressing lentiviral production

The NKG2D-4-1BB-CD3z-CAR construct (pTRPE_NKG2D-ECD_4-1BB flex plasmid) was designed and synthesized by Song et al. [15]. Characteristic of the construct could be found at Supplementary Methods (Supplementary Material File). Lentiviral vector (NKG2D-CAR:LV) production was performed essentially as previously described [23] and titrated by serial dilution transduction in 293 T cells and analysis by flow cytometry.

### Cell isolation, culture, and CAR transduction

PB mononuclear cells (PBMC) were isolated by centrifugation over a density gradient. Then PBMCs were activated and expanded for 10 days, achieving NKAE-cell expansion by co-culture with irradiated Clone9.mbIL21 feeder cells in RPMI medium supplemented with 10% human AB serum (Sigma–Aldrich, St. Louis, MO, USA) plus 100 IU/mL IL-2 (Miltenyi Biotec, Bergisch Gladbach, Germany), as previously described [4]. NKAE cells were isolated from NKAE cultures using an NK Cell Isolation Kit (Miltenyi Biotec), following the manufacturer’s instructions, and were seeded at 2 × 10^6^ cells/mL. The following day, transduction was performed with a multiplicity of infection (MOI) of 5, on plates coated with RetroNectin® (Takara Bio, Mountain View, CA, USA). Two days later, cells were harvested for experiments.

CD45RA^+^ cells were depleted by labelling of PBMCs with CD45RA microbeads (Miltenyi Biotec) for magnetic cell separation according to the manufacturer’s instructions. Purified CD45RA^−^ cells were primed at 2 × 10^6^ cells/mL overnight in X-VIVO-15 (Lonza, Basel, Switzerland) supplemented with 250 IU/mL IL-2, 10 ng/mL anti-CD3 (OKT3), and 10 ng/mL anti-CD28 antibody (28.2) (BioLegend, San Diego, CA, USA). The following day, transduction (MOI = 5) was performed on RetroNectin®-coated plates. After 10 days, the cells were harvested for experiments.

### Immunophenotyping

Flow cytometry was performed on a BD FacsCanto II™ cytometer (BD Biosciences, San Jose, CA, USA). Supplemental Table S2 lists the fluorochrome-labeled monoclonal antibodies used and the gating strategy is reported in supplemental Fig. S1. Data were analyzed using FCS Express 6 software (De Novo Software, Pasadena, CA, USA).

### Genomic stability assays

To exclude chromosomal aberrations in CAR-NK cells and NKG2D-CAR-T cells, we performed comparative genome hybridization (CGH) arrays using the KaryoNIM® STEM CELLS platform (NIMGenetics®, Madrid, Spain). DNA from samples and commercial reference sex-matched DNA (Promega Biotech) were hybridized. Bioinformatic analysis was performed using the genomic construct hg19 and Aberration Detection Method (ADM-2; window, 0.5 Mb; A = 6). The minimum number of consecutive alterations was set as 5. Duplications or deletions related to the genomic instability and anomalous proliferation of 407 genes were analyzed according to the Cancer Gene Census list (http://www.sanger.ac.uk/genetics/CGP/Census/).

### Cytokine release assay

The cytokine release profile was analyzed using the Legendplex CD8/NK-cell panel from Biolegend according to the manufacturer’s protocol. Effector and target cells were incubated at a 16:1 ratio for 4 h. Then, supernatants were harvested and analyzed using a BD FACS Canto II™ flow cytometer and BD FCAP Array™ software v.3.

### In vitro cytotoxicity assay

The cytotoxicity of NKAE and T-cell products against MM cells was assessed by Eu-TDA release assays (Perkin Elmer, AD0116, Waltham, MA, USA) performed as previously described [4]. U-266, L-363, and OPM-2 myeloma cells and primary MM plasma cells were used as target cells, and incubated for 4 h with effector cells at the indicated effector:target (E:T) ratio. To test the safety of CAR-transduced effector cells, cytotoxicity assays were performed using donor PBMC, CCD-18Co, and NL-20 cell lines as targets.

### Colony-forming cell assay

Colony-forming assays were performed to evaluate NKAE and CAR-NKAE-cell cytotoxicity against clonogenic MM cells [24]. MM cells were co-cultured with effectors cells at different E:T ratios or cultured alone for 4 h (37 °C, 5% CO_2_). MM cells were suspended in methylcellulose (Stem Cell Technologies, Vancouver, Canada) and seeded in triplicate, in a humidified chamber. Plates were incubated for 14 days (37 °C, 5% CO_2_), and colonies were counted. Representative images were acquired with a G:BOX Chemi XX6 transilluminator and analysis was performed using GeneSys image acquisition software (Syngene, Synoptics, Cambridge, United Kingdom).

### Time-lapse microscopy

Cells were prepared as described in the Supplementary Methods (Supplementary Material File). On a microfluidic system (Elveflow Plug and Play Microfluidics, Microfluidics, Roubaix, France) a constant flux of CAR-NKAE or CAR-T cells was established over 20 min in the channels with adhered MM cells. Finally, a 3 h flux of RPMI medium was performed. Cells were analyzed using a Leica AF6000W microscope (Leica Microsystems CMS GmbH, Wetzlar, Germany).

### In vivo model

An in vivo mouse model was generated using GFP-luc-expressing U-266 myeloma cells (U-266-GFP-luc). Non-obese diabetic (NOD) Cg-*Prkdc*^*scid*^ *Il2rg*^*tm1Wjl*^/SzJ (NSG) male mice (8–10 weeks old; Jackson Laboratory, Bar Harbor, ME, USA) were irradiated (1.5 Gy) and intravenously injected with either 5 × 10^6^ U-266-GFP-luc cells or PBS (Control). Three days later, the mice were infused with one single injection of 15 × 10^6^ of untransduced NKAE cells, CD45RA^−^ T cells, NKG2D-CAR-expressing NKAE cells, or NKG2D-CAR-T cells via intravenous tail injection. Mice were divided into five groups: group 1 received 5 × 10^6^ MM cells; group 2 received 5 × 10^6^ MM cells and 15 × 10^6^ untransduced NKAE cells; group 3 received 5 × 10^6^ MM cells and 15 × 10^6^ NKG2D-CAR-NKAE cells; group 4 received 5 × 10^6^ MM cells and 15 × 10^6^ CD45RA^−^ T cells; and group 5 received 5 × 10^6^ MM cells and 15 × 10^6^ CD45RA^−^ CAR-T cells. Tumor progression was evaluated every 2 weeks using the In-Vivo Xtreme Preclinical Optical/X-ray Imaging System (Bruker Sciences, Billerica, MA, USA), with imaging beginning 5 min after intraperitoneal injection of an aqueous solution of D-luciferin potassium salt (200 mg/kg) (Supplemental Fig. S2). The mice were euthanized after appearance of MM symptoms, such as asthenia, paraplegia, and weight loss of ≤20%. Weight was monitored every 2 weeks. At the endpoint, survival was registered, and tissue samples were collected. Tumor burden in BM, effector cells persistence in PB, migration to the bone marrow, and phenotype were analyzed by flow cytometry. Tissue (lung, liver, intestine) sections were fixed and stained with hematoxylin and eosin. Neither randomization nor blinding was done for animal studies.

### RNA isolation and RNA-seq

Ribosomal RNA was depleted with the NEBNext rRNA Depletion Kit (E6310L) and RNA-seq libraries were prepared using the NEBNext Ultra II Directional RNA Library Prep Kit for Illumina (E7760S) following manufacturer´s instructions. Reads were sequenced in paired-end fashion (76 bp × 2) in a NextSeq 550 sequencer, with a High Output v2 kit. Adapters and remaining ribosomal sequences were removed with bbduk (http://sourceforge.net/projects/bbmap/). The resulting reads were analyzed with Nextpresso pipeline as described in the supplementary methods (Supplementary Material File).

### Statistical analysis

Results are shown as mean ± standard error of the mean (SEM). Flow cytometry data are presented as median and interquartile range (IQR). Normal distribution and homoscedasticity were confirmed. Then, assay data were compared using the Student’s *t*-test or ANOVA. Student’s paired *t*-test or repeated measures ANOVA was used to compare cells from the same patient. In the mouse model, survival was estimated by the univariate Kaplan–Meier method, and compared using the log-rank test. Analyses were performed using SPSS v.25. Statistical significance was defined as *P* ≤ 0.05. A minimum of three replicates were performed for each experiment. For original data please contact aleivas@h12o.es.

### Ethics approval

All patients and healthy donors provided written informed consent in accordance with the Declaration of Helsinki, and the study was approved by the Hospital Universitario 12 de Octubre Institutional Review Board.

Mice were maintained in pathogen-free conditions and experiments were performed in accordance with the EU, CIEMAT and Instituto de Investigación Sanitaria Hospital Universitario 12 de Octubre guidelines upon approval of the protocols by the Environment Department in Comunidad de Madrid, Spain (PROEX 191.2/20).

## Results

### NKG2D ligands are expressed in multiple myeloma cells

**Fig. 1:**
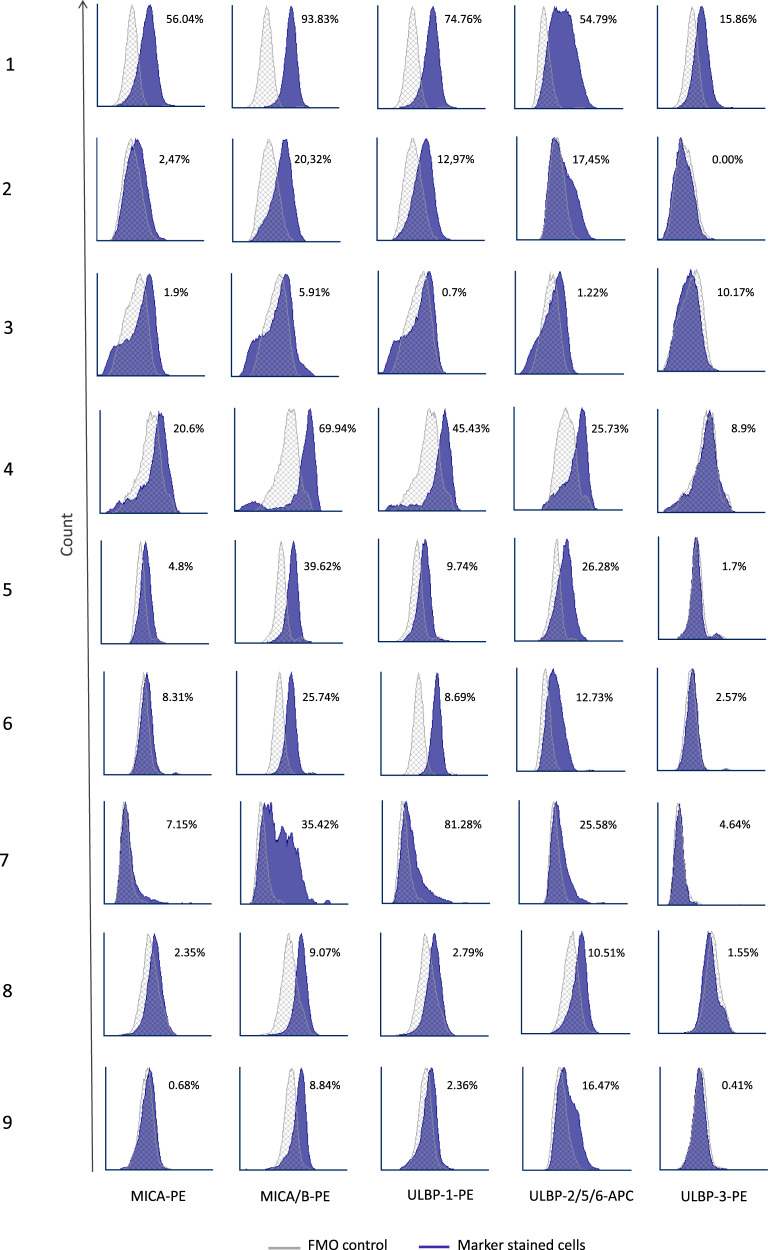
NKG2D ligand expression in MM cells. Expressions of MICA, MICA/B, ULBP-1, ULBP-2/5/6, and ULBP-3 were analyzed in plasma cells from bone marrow samples of MM patients. Representative histograms are shown. For each staining, the percentages of positive cells versus the fluorescence minus one (FMO) control are indicated.

We analyzed samples from 9 MM patients and 9 MM cell lines for surface expression of NKG2D ligands (MICA, MICB, ULBP-1, ULBP-2/5/6, and ULBP-3). NKG2DL expression widely varied among patients’ samples (Fig. 1). MM cell lines showed high NKG2DL expression, except for ULBP-2/5/6 (10.7% ± 5.48%) (Supplemental Figs. S3 and S4A).

Compared to cell lines, MM samples exhibited lower expression of MICA and ULBP-3. Defining positive expression as >5% positive cells for one marker, 44.4% of patients were positive for MICA, 100% for MICA/B, 66.7% for ULBP-1, 88.9% for ULBP-2, and 22.2% for ULBP-3. Patient MM cells widely varied in expressions of MICA/B (93.8–5.91%) and ULBP-1 (81.28–0.7%) (Supplemental Fig. S4B).

### NKAE cells and memory T cells from MM patients could be transduced with a NKG2D-CAR

NKAE cells and CD45RA^−^ memory T cells were transduced using NKG2D-CAR:LV at a low MOI [5]. We compared lentiviral transduction efficiency between paired NKAE and CD45RA^−^ memory T cells. Both cell types were purified before transduction, and verified as ≥98% pure by flow cytometry (Supplemental Fig. S1).

**Fig. 2:**
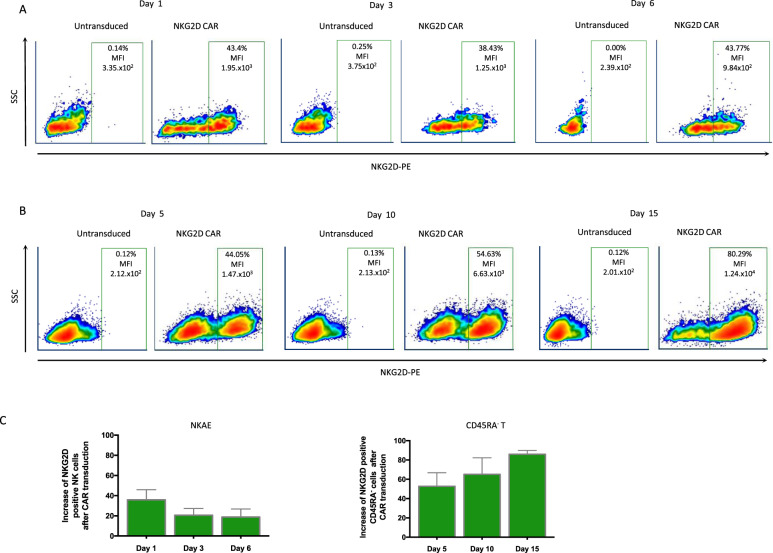
NKG2D-CAR transduction. NKAE cells and CD45RA^−^ T cells were transduced with an NKG2D-CAR. Representative dotplots are shown for NKAE cells (**A**) and CD45RA^−^ T cells (**B**) transduction. Both cell types showed increased percentages of NKG2D-positive cells (over the percentage of untransduced cells) and median fluorescence intensity (MFI). The expression remained stable in CAR-expressing NKAE cells after 6 days; however, T cells showed more stable transduction (**C**). Results are shown as the mean ± SEM of five different experiments.

NKAE cells showed high endogenous NKG2D expression. Thus, we analyzed NKG2D expression due to NKG2D-CAR:LV transduction using endogenous expression as baseline (paired untransduced NKAE cells were also seeded in Retronectin®-coated plates). At 24 h post-transduction, CAR-NKAE cells showed an increased percentage of NKG2D-positive cells and increased median fluorescence intensity (MFI) (Fig. 2A). However, after 3 days, NKG2D expression decreased by 15.25% (Fig. 2A). Then, CAR-NKAE cells suffered only a minimal loss of NKG2D expression at 6 days post-transduction (transduction efficiency 20.65% ± 5.95). CD45RA^−^ memory T cells showed higher transduction efficiency (52.68% ± 12.2) at 5 days post-transduction. Compared to NKAE cells, memory T cells were more efficiently and stably transduced, and maintained increased NKG2D expression even up to 15 days post-transduction (Fig. 2B-C).

### CAR-transduced cells preserve their phenotype and chromosomal stability after CAR transduction

**Fig. 3:**
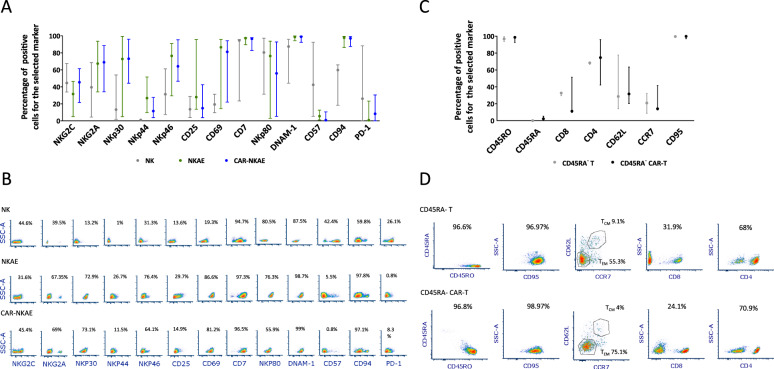
Phenotype of NKAE cells and T cells. The expression profile of different surface markers was analyzed by flow cytometry in untransduced and CAR-expressing (**A**) NKAE cells and (**C**) T cells. Data are represented as median and IQR of three different experiments. Statistical analysis was performed for each pair of untransduced and CAR-transduced cells. Representative density plots of (**B**) NK cells, NKAE cells, and CAR-NKAE cells and density plots of (**D**) CD45RA^−^ T cells and CD45RA^−^ CAR-T cells are shown.

NKAE cells and CAR-NKAE cells showed a highly activated phenotype when compared to unstimulated NK cells. The expression profiles did not significantly differ between paired untransduced NKAE cells and CAR-NKAE cells and between CD45RA^−^ T cells and CD45RA^−^ CAR-T cells (Fig. 3).

CGH arrays were performed and no difference was detected between untransduced and CAR-transduced cells, indicating that CAR transduction did not adversely affect the genomic stability of NKAE cells (raw data are available in a public repository: “Raw data GEO OMNIBUS 19ID00315-318”, Mendeley Data, V1, 10.17632/4cpvmbcj43.1).

### NKG2D-CAR-NKAE cells eradicate MM cells

NKAE cells showed robust cytotoxic activity against the U-266 MM cell line, and destroyed 77.7% of MM cells at the maximum E:T ratio tested (32:1). Compared to untransduced NKAE cells, CAR-NKAE cells exhibited improved cytotoxicity, destroying 100% of MM cells at a lower E:T ratio (8:1) (Supplemental Fig. S5).

**Fig. 4:**
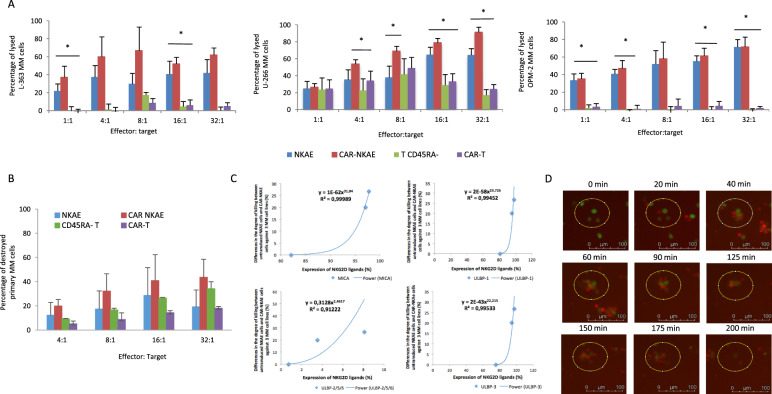
Antimyeloma activity of CAR-NKAE and memory CAR-T-cell products. **A** Cytotoxic activity of untransduced NKAE cells and memory T cells (CD45RA^−^ T cells), CAR-NKAE cells, and memory CAR-T cells against different MM cell lines. Results are shown as mean ± SEM of four independent experiments. *P < 0.05 compared to untransduced cells (NKAE and memory T cells) and memory CAR-T cells. **B** Cytotoxicity of NKAE and CAR-NKAE against primary MM cells from bone marrow sample at different E:T ratios (triplicates). **C** Plots representing the relationship between the differences in the degree of killing (between NKAE cells and CAR-NKAE cells) and the expression of NKG2DL (MICA, ULBP-1, ULBP-2/5/6, and ULBP-3) in 3 MM cell lines with different expression of NKG2DL. **D** Time-lapse experiments were performed, U-266 MM cells (green) were destroyed by CAR-NKAE cells (red) with only 20 min of exposure. After 3 h, MM cells were destroyed and released the green fluorescent probe.

Memory T cells lack of a reactive T-cell receptor and maintain T-cell immunity producing less CRS [22, 25]. For this reason, the ability of CAR-NKAE cells to target MM cells was compared to those of memory CAR-T cells from MM patients. Compared to untransduced NKAE cells, memory T cells and CAR-T cells, CAR-NKAE cells exhibited enhanced cytotoxicity against MM cell lines (Fig. 4A-B).

As expected, NK and T cells demonstrated the best cytotoxic activity against U-266 cells, which exhibited the highest expression of NKG2D ligands (>97% for MICA, MICA/B, ULBP-1, ULBP-3, and 8% ULBP-2/5/6) when compared to OPM-2 (75–95% for MICA, MICA/B, ULBP-1, ULBP-3, and 0.73% for ULBP-2/5/6) or L-363 (94–97% for MICA, MICA/B, ULBP-1, ULBP-3, and 3.53% for ULBP-2/5/6) (Fig. 4A-C).

Moreover, differences in the degree of killing between untransduced NKAE cells and CAR-NKAE were higher against U-266 and L-363 cells than against OPM-2 cells, which could be related with the lower degree of expression of NKG2D ligands in the OPM-2 cell line: MICA (R^2^ = 0.99989), ULBP-1 (R^2^ = 0.99452), ULBP-2/5/6 (R^2^ = 0.91222), and ULBP-3 (R^2^ = 0.99533) (Fig. 4C).

Time-lapse microscopy confirmed CAR-NKAE cells antimyeloma activity and revealed that CAR-NKAE cells had equal migration and adhesion capacity compared to memory CAR-T cells and NKAE (Fig. 4D and supplemental video could be found in a public repository “Time-lapse microscopy of CAR-NKAE cells and memory CAR-T cells from multiple myeloma patients”, Mendeley Data, V1, 10.17632/m79ygj3rwr.1”).

**Fig. 5:**
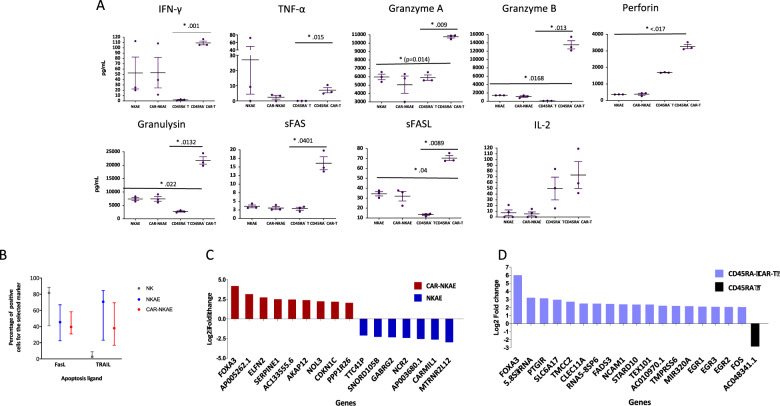
Death cell mechanisms of NKAE and T-cell products. **A** Cytokines and cytotoxic molecules release by NKAE cells, CAR-NKAE cells, memory T cells, and memory CAR-T cells. **B** Expression of apoptosis ligands: FasL and TRAIL, and CD16 (antibody-dependent cell-mediated cytotoxicity) and representative flow cytometry density plots. RNA-seq and differential gene expression analysis were performed in **C** NKAE/CAR-NKAE cells and **D** T /CAR-T cells.

Mechanisms of cell death have been analyzed. NKAE and T cells secreted all the analyzed cytokines. However, CAR-T cells exhibited higher production of IFN-γ (*p* = 0.001), TNF-α (*p* = 0.015), granzyme A (*p* = 0.009), granzyme B (*p* = 0.013), perforin (*p* = 0.0176), granulysin (*p* = 0.0132), sFas (*p* = 0.0401), and sFasL (*p* = 0.022) than untransduced T cells (Fig. 5A). The expression of cell apoptosis ligands exhibits an increase of TRAIL expression and a decrease in FasL expression in NKAE cells and CAR-NKAE when compared to NK cells (Fig. 5B). Differential gene expression analysis reveals an overexpression of FOXA3 in both CAR-NKAE cells and CAR-T cells when compared to their untransduced counterparts (Fig. 5C and D). Gene ontology analysis showed that CAR-NKAE exhibited overexpression of genes involved in cell activation (FDR 0.008), migration (FDR 0.02), exocytosis (FDR 0.015), and immune effector process (FDR 0.037). CAR-T cells overexpressed genes involved in response to cytokine (FDR 0.0), cell activation (FDR 0.0), immune effector process (FDR 0.0), inflammatory response (FDR 0.002), and T-cell activation (FDR 0.005). RNA-seq data are available under the GEO accession GSE179408.

### NKG2D-CAR-NKAE cells destroy clonogenic MM cells

**Fig. 6:**
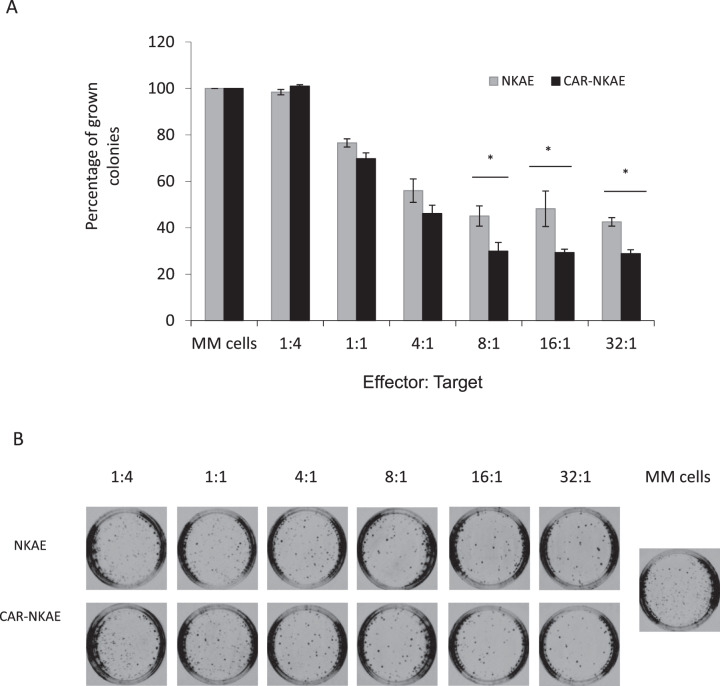
CAR-NKAE reduces clonogenicity of MM. **A** L-363 MM cells were exposed to different concentrations of NKAE cells or CAR-NKAE cells (E:T ratios of 1:4 to 32:1) and then seeded in methylcellulose to evaluate the cytotoxicity against clonogenic MM cells. Results are shown as percentage of colonies grown (with autonomous MM growth set as 100%), and represented as mean ± SEM of triplicates. *P < 0.05. **B** Representative pictures of colonies grown at different E:T ratios.

Compared to NKAE cells, CAR-NKAE showed better reduction of clonogenicity than NKAE cells against MM clonogenic tumor cells [26]. We found significant differences in killing ability at E:T ratios at or above 8:1 (*P* = 0.039) (Fig. 6).

### NKG2D-CAR-NKAE cells display potent efficacy in vivo

**Fig. 7:**
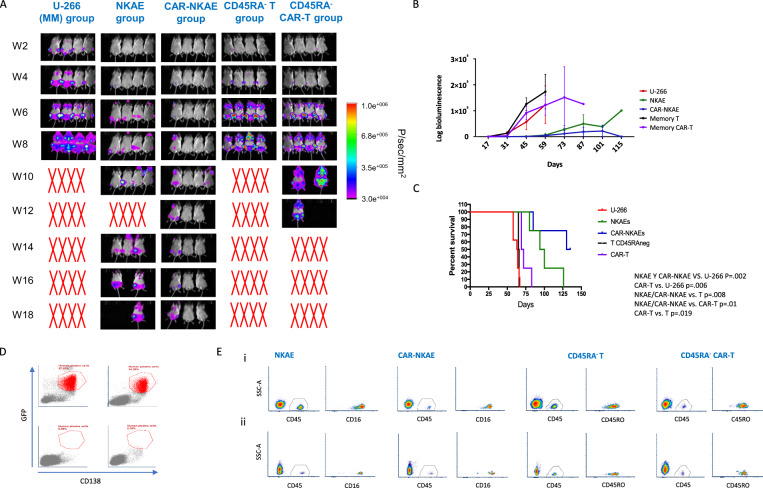
NKG2D-CAR-transduced NKAE cells exhibit potent efficacy in vivo. **A** Imaging of tumor burden monitored by bioluminescence at the indicated timepoints in MM mice, NKAE-cell-treated mice, and CAR-NKAE-cell-treated mice (At day 73 mice 2 and 3 from NKAE group were accidentally interchanged). **B** Quantification of the bioluminescence signal in MM mice, NKAE-cell-treated mice, CAR-NKAE-cell-treated, CD45RA^−^ T-cell-treated mice and CD45RA^−^ T-cell-treated mice at the indicated timepoints and **C** Kaplan–Meier survival curves. **D** Representative flow cytometry dotplots showing bone marrow infiltration (GFP^+^ CD138^+^) of MM mice (top left), NKAE-cell-treated mice (top right), and the two CAR-NKAE-treated mice that were disease free at the end of the study (bottom). **E** Persistence of effector cells in peripheral blood (i) 10 days after infusion and (ii) 20 days after infusion. Representative flow cytometry density plots for NKAE/CAR-NKAE cells that were identified by human CD45 and CD16 labelling and for CD45RA^−^ T/CAR-T cells that were identified by human CD45 and CD45RO labelling.

Mice receiving NKAE cells and CAR-NKAE cells survived twice as long as mice in the untreated control group (MM group). However, mice receiving memory T cells or CAR-T cells survived as long as the untreated group (Fig. 7A and 7C) and those receiving CAR-T cells survived for only 20 more days. The MM group died due to tumor burden 60 days after MM cell injection, showing MM clinical signs and high bioluminescent signal (Fig. 7B). Mice receiving NKAE cells (NKAE group) showed extended survival, with half of mice dying 100 days after treatment administration. Only those mice receiving CAR-NKAE cells survived until the end of the experiment (127 days after treatment, 129 after MM cells infusion), and 25% (2/8) of them remained disease free with no bioluminescence signal (Fig. 7A and B) Flow cytometry for GFP-positive and CD138-positive cells in the bone marrow of these mice revealed no plasma cells (0.00%) or very small numbers (0.05%) of plasma cells (Fig. 7D).

Effector cells (human CD45^+^ cells, CD56^+^ CD16^+^ NK cells and CD45RO^+^ memory T cells) were detected in PB 10 days after infusion: NKAE cells (17.3% ± 3.97) and memory T cells (11.31% ± 0.92). CAR-transduced cells were also detected; however, persistence was lower than that of untransduced cells: CAR-NKAE cells (3.51% ± 1.26) and CAR-T cells (1.49% ± 0.49). Although a decrease was observed, NKAE were able to persist until 20 days in PB (0.82% ± 0.1) after infusion as well as CAR-NKAE cells (0.16% ± 0.03), memory T cells (3.55% ± 0.72), and CAR-T cells (0.78% ± 0.2) (Fig. 7E). An effector memory (EM) phenotype (CD45RO^hi^, CD62^lo^, and CCR7^lo^) was observed for untransduced T cells (46.88% ± 1.85) and CAR-T cells (46.6% ± 3.2).

To analyze the migratory potential of the cells, flow cytometry of mice bone marrow was performed. NKAE cells and T cells were detected into the bone marrow 7 days and 14 days after infusion, respectively (Supplemental Fig. S6A). However, NKAE and CAR-NKAE cells (0.29% ± 0.07 and 0.12% ± 0.02, respectively) were detected in a lower percentage than T and CAR-T cells (1.85% ± 0.17 and 1.76% ± 0.38, respectively). Migratory NKAE cells exhibited an activated phenotype with high expression of costimulatory molecules like NKp80 and DNAM-1, and activation receptors like CD25. Migratory CAR-NKAE cells exhibited higher expression of NKG2D than NKAE cells (MFI 3549 ± 192 A.U. vs. 1881 ± 32 A.U.) and upregulation of CD57 and apoptosis ligands like FasL (17.02% ± 2.3 vs. 6.5% ± 0.9) indicating a highly cytotoxic phenotype (Supplemental fig. S6B). Migratory CD45RA^−^ T cells were mostly CD4^+^ (95.65% ± 0.46 and 93.3% ± 0.07) and exhibited a T_EM_ phenotype, 74.45% ± 6.4 and 87.95% ± 1.02 for T and CAR-T cells, respectively.

### NKG2D-CAR-expressing NKAE cells are safe in vitro and in vivo

**Fig. 8:**
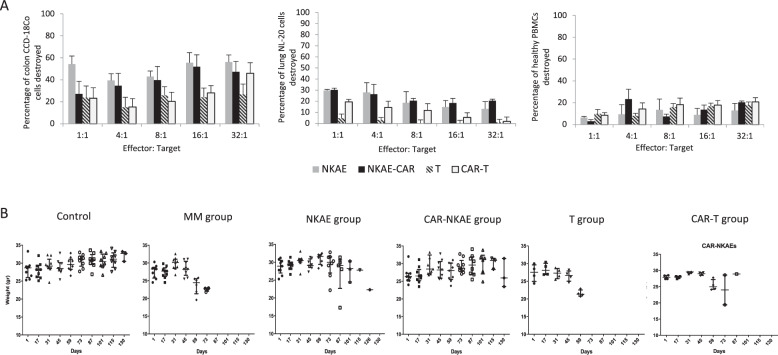
Toxicity of CAR-expressing cells. **A** The cytotoxicity of CAR-NKAE and CAR-T cells against healthy tissues (lung, colon, and PBMCs) was evaluated and compared to that of untransduced NKAE and T cells. Results are represented as mean ± SEM of four replicates. **B** In vivo toxicity was evaluated by monitoring the weights of mice treated during treatment.

Healthy cells from NL-20 (lung) and CCD-18Co (colon) cell lines and allogeneic PBMCs, with a basal expression of NKG2D ligands, were exposed to CAR-transduced NKAE cells. CAR-NKAE cells showed low cytotoxicity against lung cells and PBMCs, and higher-than-expected cytotoxicity against colon cells. Untransduced NKAE cells and CAR-NKAE cells exhibited similar toxicity profiles (Fig. 8A).

CAR-T-cell therapy usually produces graft vs. host disease (GvHD) roughly within two months of treatment [27]. However, CAR-NKAE cells did not produce any sign of GvHD or treatment-related toxicities during the 150 days of experiment. Weight loss was only associated with MM development in our study. Mice receiving CAR-NKAE cells exhibited no weight loss during the experiments (Fig. 8B). Mice treated with CAR-T exhibited weight loss related to disease progression. After sacrifice no hepatosplenomegaly or secondary neoplasia were found (not shown). Hematoxiline-eosin sections from tissues (spleen, liver, lung, and intestine) were analyzed and no signs of graft vs. host disease were observed in the mice treated with CAR-NKAE cells (Supplementary Fig. S7).

## Discussion

CAR cell products are a promising new weapon against MM, but challenges include finding specific tumor-associated antigens and reducing CAR-related toxicities [18]. Several reports describe the use of CAR-T cells focused on MM markers such as CD138, CS1, or BCMA, to achieve high antitumor activity and avoid off-tumor toxicities [28–30]. However, the expression of CD138 and CS1 in healthy tissues and the known toxicities and low efficacy observed with monotherapy of their monoclonal antibodies predecessors suggest that these targets may not be ideal [31, 32].

The advantage of NKG2D-CAR therapy is to achieve high activity against tumor cells regardless of cell type. All analyzed primary MM samples exhibited high expression at least of one NKG2DL, and we previously found that resistant and clonogenic MM tumor cells expressed NKG2DL [26]. Thus, in MM cases with heterogeneous expression of NKG2D ligands, immunotherapy with NKG2D-CAR-transduced cell products may lead to eradication of MM cells, even resistant cells with self-renewal potential [33]. Furthermore, NKG2DL are also expressed on immunosuppressive cells, such as regulatory T cells and myeloid-derived suppressor cells[34], making NKG2D-CAR treatment an attractive treatment option for both hematological malignancies and solid tumors [35, 36] both controlling the immunosuppressive environment and the development of the neovasculature [37].

NK-cell lines can be used to produce CAR-NK cells, representing a renewable source that enables easy attainment of large cell numbers. However, they are derived from NK-cell neoplasia, and require irradiation before infusion, which significantly reduces their persistence in vivo and their clinical efficacy [38]. We previously established a method to produce high numbers of primary NKAE cells from MM patients [4]. Here we demonstrated that these NKAE could be transduced to express NKG2D-CAR using a low MOI, thus limiting the risks of CAR therapy reducing the potential for genomic disruption or instability. NKG2D-CAR:LV transduction enhanced NKAE-cell activity and these NKGD2-CAR:LV-NKAE cells showed higher cytotoxic activity than memory T cells. Transduction process with a NKG2D-CAR:LV showed a generalized upregulation of cytotoxicity mechanisms in T cells with increased release of cytokines, lytic granules as well as soluble Fas/FasL. CAR-NKAE cells, exhibited an increase in the expression of genes involved in the processes of immunological activation.

Memory T cells from MM patients were easily expanded and transduced with NKG2D-CAR, but showed weak cytotoxicity compared to CAR-NKAE-cell products, suggesting that autologous memory CAR-T cells therapy may not be effective in MM. Several methods can be used to produce allogeneic memory CAR-T cells (CD45RA^−^ T cells) expressing NKG2D [39]. Allogeneic memory CAR-T from healthy donors reportedly exhibited potent cytotoxicity against osteosarcoma cells [16]. It has been previously described that T cells, like NK cells, exhibit a dysregulation affecting their cytotoxic activity in MM patients [2]. However, here we report a robust expansion and activation process for NK cells combining co-culture with feeder cells and CAR transduction, which is capable to overcome this impairment.

There is previous evidence that justifies the use of CAR-NK cells for the treatment of hematological malignancies. Prior studies have shown that NK cells from PB can be stably transduced with a retroviral vector against CD19^+^ tumors reaching high transduction efficiency [40] and that these cells are effective in a clinical setting lacking of serious adverse effects [41].

Two prior studies report the use of PB NK cells expressing NKG2D-CAR. Chang et al. designed a second-generation NKG2D-CAR and efficiently transduced it into previously expanded allogeneic NK cells. However, it is difficult to compare these CARs, as they have different costimulatory domains (DAP10 vs. 4-1BB) and different cloning vectors (retrovirus vs. lentivirus). Chang et al. tested the cytotoxic activity against hematological cell lines, but not against MM, and found high cytotoxicity [42]. In a xenograft murine model of osteosarcoma, tumor burden reduction was achieved using NKG2D-CAR-NK cells, but all mice eventually developed disease [43]. The use of 4-1BB costimulatory domain in CAR constructs has demonstrated to potentiate the in vitro activity of T cells, NK cells, and other immune effectors [44], and we have previously demonstrated that stimulation of NK cells with 4-1BBL (CD137L) modified feeder cells enhanced the activity of NK cells [4]. The merging of two different stimulation ways with 4-1BB could be at the basis for the strong cytotoxic activity found in NKG2D-CAR-NKAE cells.

Xiao et al. employed NKG2D-CAR-expressing NK cells generated by RNA electroporation, containing DAP12 or CD3ζ as the signaling domain [45]. RNA electroporation is a simple and reliable method [46], but it provides maximum transduction efficiency at 24 h, which is quickly lost over subsequent days [47]. Notably, multiple CAR-NK-cell injections in tumor-bearing mice were needed to delay disease progression [45]. Lentiviral transduction procures more stable transduction. However, transduction of primary autologous NK cells remains challenging. Other transduction strategies for NKAE cells need to be explored in the future like transduction with lentiviral vectors pseudotyped with Baboon envelope [48, 49] or the use of antiviral defense mechanisms inhibitors [50]. Transposon constructs are an interesting strategy [51]; however, two cases of CAR-positive lymphoma have recently been reported [52].

Multiple studies describe engineering CAR-NK cells from NK-cell lines expressing NKG2D. NK-92 is most commonly used since it shows reproducible high cytotoxicity [53]. Compared with other activating receptors, NKG2D-CAR-NK-92 cells exhibited and equal cytotoxic potential [54]. However, they have limited survival and proliferation due to irradiation. The activity of NKG2D-CAR-NK-92 cells against MM is yet to be explored [55].

Patient-derived NKG2D-CAR-T cells show efficacy against MM in vitro and in vivo using an NKG2D-CAR comprising the NKG2D receptor extracellular domain fused with CD3ζ [56, 57]. NKG2D-CAR-T cells reportedly display lethal toxicity in an in vivo mouse model, with acute toxicity similar. However, was strain-dependent and CAR construct-dependent [58]. NK-cell safety has already been verified against different malignancies [59, 60]. Our group previously reported that NKAE lacked toxicity in a phase I clinical trial of MM [4]. Our present experiments revealed some toxicity against two cell lines. However, immortalized cell lines are not truly representative of healthy tissues, as they have been genetically manipulated to persist in vitro. CAR-NKAE cells displayed the same toxicity profile as NKAE cells. Nevertheless, in our in vivo model, no toxicity was observed in those mice treated with NKG2D-CAR-NKAE cells. Xenograft models using immunocompromised mice for MM are useful to evaluate the direct cytotoxic effect of CAR-redirected effector cells on cancer cells; however, they cannot be used to explore the adaptive immune response elicited by CAR-based immunotherapies.

In summary, this is the first report demonstrating the reproducibility and feasibility of using autologous NKAE cells bearing NKG2D-CAR to treat MM and showing that CAR-NKAE cells are a better strategy against MM than memory CAR-T cells. We also demonstrated that merging ex vivo expansion and activation of primary NK cells and NKG2D-CAR transduction is a feasible approach. NKAE cells were transduced and NKG2D-CAR transduction enhanced the potent antitumor activity both in vitro an in vivo. While memory T cells are also susceptible to CAR transduction, they were not effective against MM in an autologous setting. In contrast, autologous CAR-NKAE cells displayed strong cytotoxic activity with no signs of toxicities, and completely abrogated MM growth in a mouse model. These results support the use of autologous NKG2D-CAR-expressing NKAE cells as a treatment for refractory MM.

## Supplementary information

Supplemental material
