# Supplementary material for: NKG2D-CAR-transduced natural killer cells efficiently target multiple myeloma
Source: Blood Cancer J. 2021 Aug 14;11(8):146. doi: 10.1038/s41408-021-00537-w (PMC8364555; doi:10.1038/s41408-021-00537-w)
Supplement: Supplementary file 1 — Supplemental material [file 41408_2021_537_MOESM1_ESM.docx]

**NKG2D-CAR-transduced natural killer cells efficiently target multiple myeloma**

**Supplementary Material**

**Supplementary Methods**

**Cell lines**

NL-20 normal human bronchial epithelial cells (CRL-2503), CCD-18Co normal human colon fibroblasts (CRL1459), MM.1S (CRL-2974) and MM.1R (CRL-2975) cell lines were obtained from ATCC® (Manassas, VA, USA). U-266 (ACC-9), L-363 (ACC-49), OPM-2 (ACC-50), NCI-H929 (ACC-163), JJN-3 (ACC-541), RPMI-8826 (ACC-402), and SK-MM-2 (ACC-430) myeloma cells were purchased from the DSMZ (Braunschweig, Germany). Cells were cultured in RPMI-1640 medium (Biowest, Nuaillé, France) with 10% FBS (Hyclone, GE Healthcare, Little Chalfont, United Kingdom) in a humidified 37°C chamber in 5% CO_2_.

**CAR-expressing lentiviral production**

The NKG2D-4-1BB-CD3z-CAR construct was cloned into a third-generation lentiviral backbone, under regulation of a EF1-α promoter bearing the extracellular portion of NKG2D (amino acids 82-216), a CD8 transmembrane hinge, and the costimulatory and signaling domains 4-1BB and CD3ζ. HEK293T cells were transiently transfected with the transfer plasmid and lentiviral helper plasmids pMDLg-pRRE (Gag/Pol expression plasmid), pRSV.REV (Rev expression plasmid), pMD2.VSV.G (VSV expression plasmid), and pAdvantage (Promega Biotech, Wisconsin, USA).

**Time-lapse microscopy**

U-266 MM cells were stained for 30 min with CFDA/SE 0.25 μM (Vybrant CFDA/SE Cell Tracer Kit, V12883, Thermo Fisher Scientific, Waltham, MA, USA) and seeded at 1 × 10^6^ cells/ml on a six-channel poly-L-lysine (Sigma-Aldrich)-treated flow chamber. The cells formed a monolayer, and were incubated for 30 min in a humidified chamber (37°C, 5% CO_2_). NK cells were stained with 0.5 μM CellTracker™ Orange CMTMR Dye (C2927, Invitrogen, Waltham, MA, USA) in serum-free RPMI medium, and then resuspended at 0.25 × 10^6^ cells/mL in RPMI plus 10% FBS. Then a constant flux of CAR-NKAR or CAR-T cells was established over 20 mins and a 4-hour RPMI medium flux was performed.

**RNA isolation and RNA-Seq**

Ribosomal RNA was depleted with the NEBNext rRNA Depletion Kit (E6310L) and RNA-seq libraries were prepared using the NEBNext Ultra II Directional RNA Library Prep Kit for Illumina (E7760S), using indexes from NEBNext® Multiplex Oligos for Illumina® (Dual Index Primers Set 1, E7600S). Reads were sequenced in paired-end fashion (76bp x 2) in a NextSeq 550 sequencer, with a High Output v2 kit. Adapters and remaining ribosomal sequences were removed with bbduk (<http://sourceforge.net/projects/bbmap/>). The resulting reads were analysed with the nextpresso pipeline as follows: sequencing quality was checked with FastQC v0.11.0 (https://www.bioinformatics.babraham.ac.uk/projects/fastqc/). Reads were aligned to the human genome (GRCh38) with TopHat2 (1) using Bowtie1 (2) and Samtools (3), allowing 3 mismatches and 20 multihits. The Gencode v37 gene annotation for GRCh38 was used. Read counts were obtained with HTSeq (4). Differential expression and normalization was performed with DESeq2 (5), keeping only those genes with more than 2 normalized counts in at least 30% of the samples. Finally, those genes that had an adjusted p-value below 0.05 FDR were selected. GSEAPreranked (6) was used to perform gene set enrichment analysis for the selected gene signatures on a pre-ranked gene list, setting 1000 gene set permutations. Only those gene sets with significant enrichment levels (FDR q-value < 0.05) were considered.

**References:**

1. Trapnell C, et al. Differential gene and transcript expression analysis of RNA-seq experiments with TopHat and Cufflinks. Nat Protoc. 2012;7(3):562-78.

2. Langmead B, Trapnell C, Pop M, Salzberg SL. Ultrafast and memory-efficient alignment of short DNA sequences to the human genome. Genome Biol. 2009;10(3):R25.

3. Li H, et al. The Sequence Alignment/Map format and SAMtools. Bioinformatics. 2009;25(16):2078-9.

4. Anders S, Pyl PT, Huber W. HTSeq--a Python framework to work with high-throughput sequencing data. Bioinformatics. 2015;31(2):166-9.

5. Love MI, Huber W, Anders S. Moderated estimation of fold change and dispersion for RNA-seq data with DESeq2. Genome Biol. 2014;15(12):550.

6. Subramanian A, et al. Gene set enrichment analysis: a knowledge-based approach for interpreting genome-wide expression profiles. Proc Natl Acad Sci U S A. 2005;102(43):15545-50.

**Supplementary Figures**

**Figure S1**

A

**
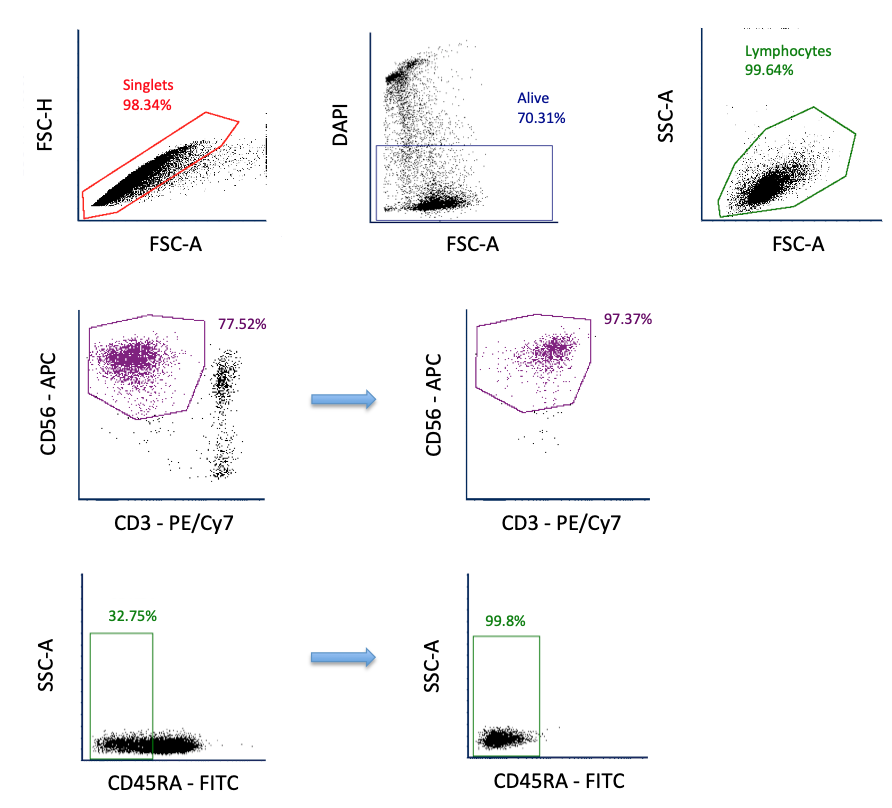
**

B

C

**Supplemental Figure S1. Cell purification before CAR transduction.** (A) Analysis strategy was based on doublets and dead cells exclusion before selection of the population of interest (lymphocytes). When possible, a minimum of 10,000 events within the population of interest were analyzed. Before CAR transduction, immunomagnetic labelling was performed to purify NKAE cells (B) and memory T cells (C).

**Figure S2**

**
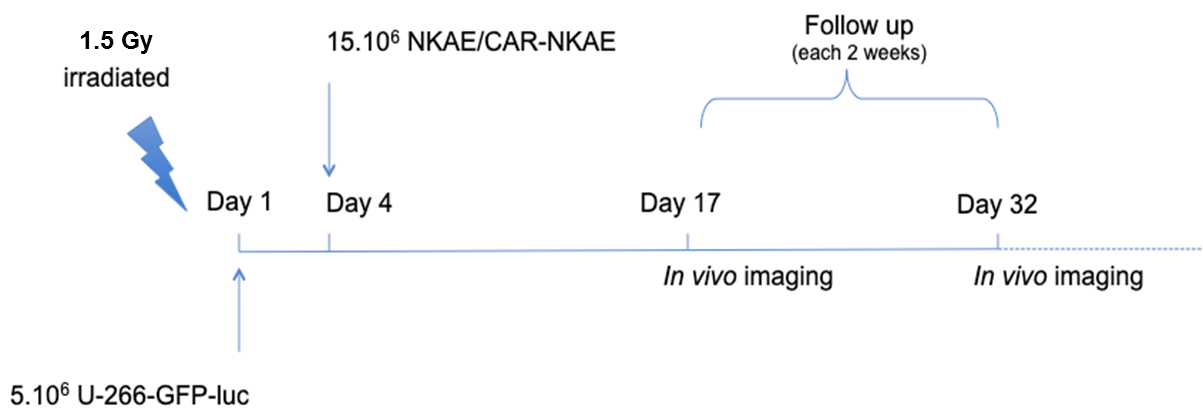
**

**Supplemental Figure S2. Schematic representation of the *in vivo* xenograft mouse model of MM.** One single injection of 5.10^6^ U-266-GFP-luc MM cells was performed at day 1. Three days later, mice received a single injection of 15.10^6^ NKAE cells, 15.10^6^ CD45RA^-^ T cells, 15.10^6^ CAR-T cells or 15.10^6^ CAR-NKAE cells. Tumor burden was monitored by bioluminescence every 15 days.

**Figure S3**


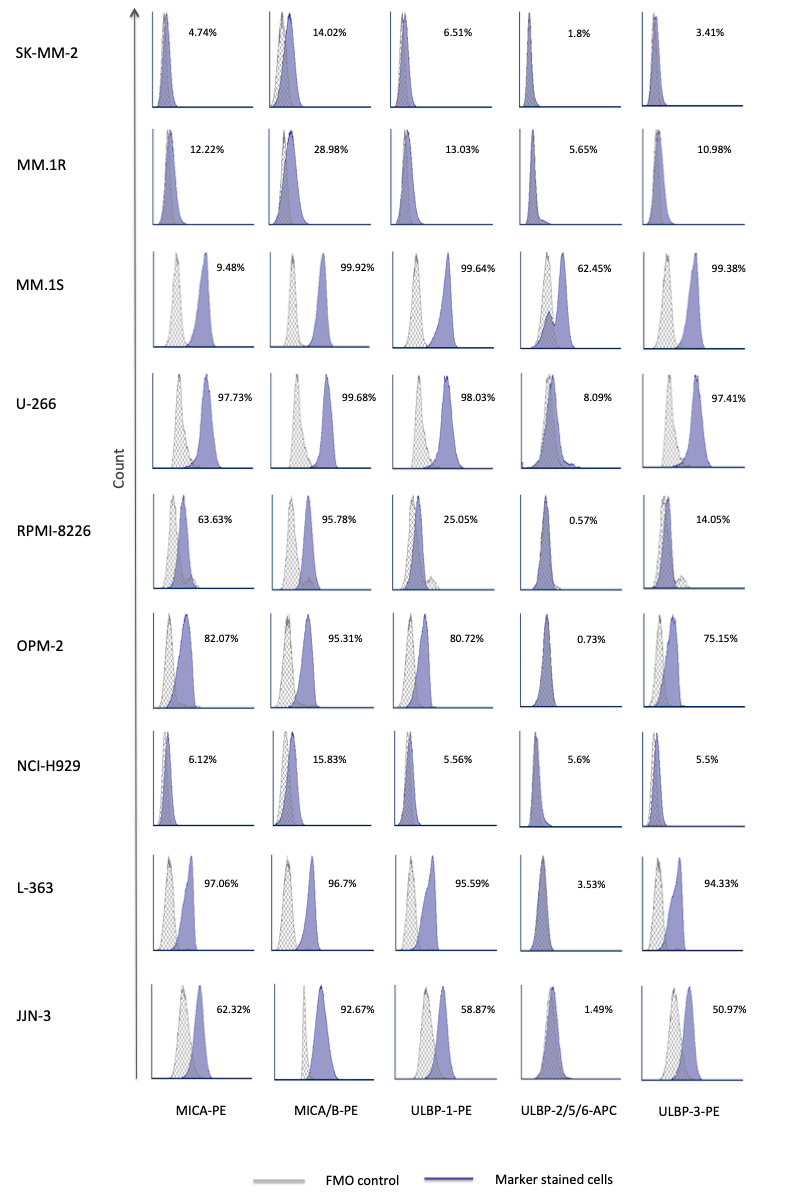


**Supplemental Figure S3. NKG2D ligand expression in MM cell lines.** Expressions of MICA, MICA/B, ULBP-1, ULBP-2/5/6, and ULBP-3 were analyzed in nine different MM cell lines. Representative histograms are shown. For each staining, the percentages of positive cells versus the fluorescence minus one (FMO) control are indicated.

**Figure S4**

A

B

**Supplemental Figure S4. Mean expression of NKG2D ligands in MM cells.** We analyzed MICA, MICA/B, ULBP-1, ULBP-2, and ULBP-3 expression in nine different MM cell lines (A) and nine primary samples (B). Results are expressed as mean ± SEM.

**Figure S5**


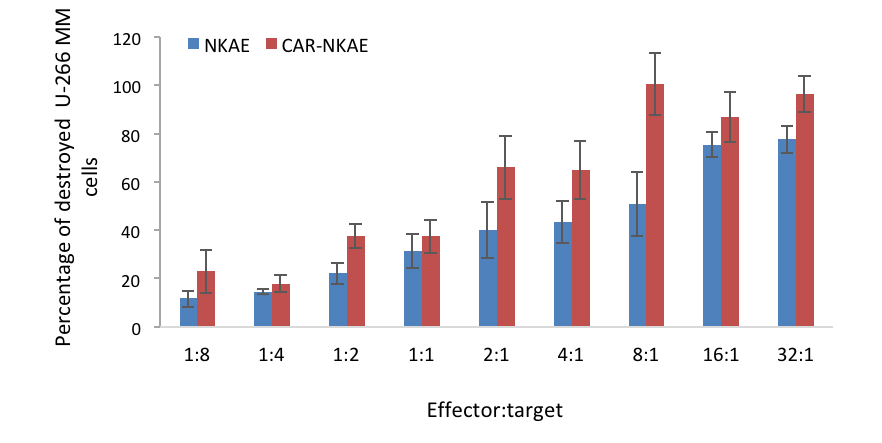


*

*

*

*

**Supplemental Figure S5**. **Anti-myeloma activity of CAR-NKAE cell products.** In vitro cytotoxicity of untransduced NKAE cells and NKG2D-CAR NKAE cells from MM patients against U-266 MM cells at different effector:target (E:T) ratios. Results are shown as mean ± SEM of 8 different experiments with different CAR-NKAE cells. * P < .05.

**Figure S6**

**
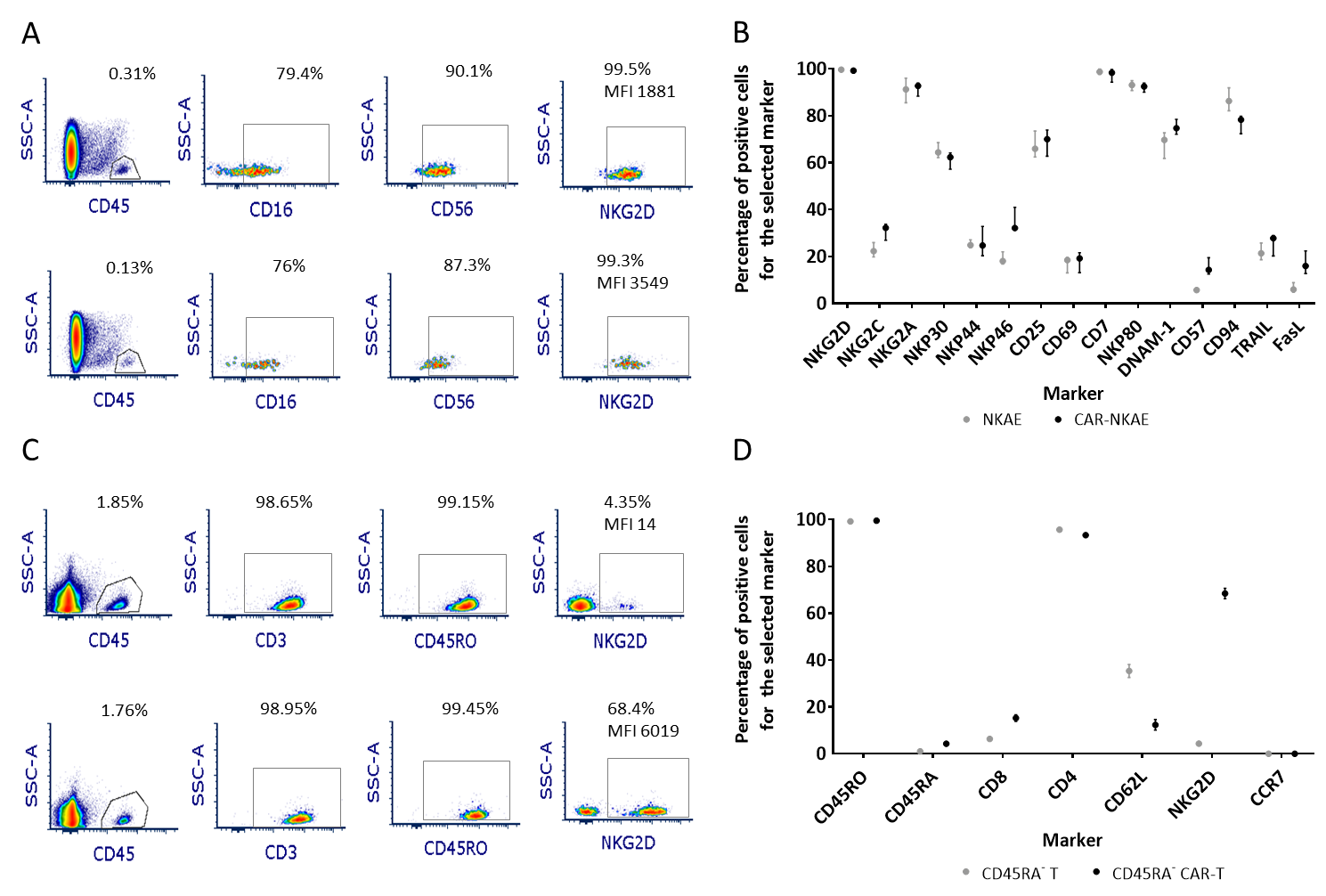
**

**Supplemental Figure S6**. ***In vivo* migratory potential of NKAE and memory T cell products.** (A) Representative flow cytometry dots plots and (B) expression profile of surface cell markers (data are represented as median and IQR of three different experiments) of the bone marrow from mice treated with NKAE/CAR-NKAE (CD45^+^CD56^+^CD16^+^) cells. (C) Representative flow cytometry dots plots and (D) expression profile of surface cell markers of T/CAR-T (CD45^+^CD3^+^CD45RO^+^) cells that were able to migrate to the bone marrow.

**Figure S7**


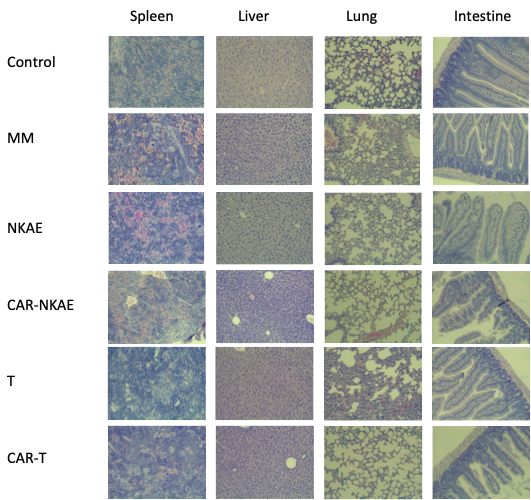


**Supplemental Figure S7**. **Toxicity against healthy tissues.** After sacrifice tissues (spleen, liver, lung and intestine) from mice treated with (B) NKAE, (C) CAR-NKAE, (D) T and (E) CAR-T cells were fixed, stained with He-Eo and analyzed. (A) Tissues from control mice without disease were used as a reference. Tissues were analyzed using a Leica DM2000 LED microscope (20X).

**Table S1**

| Patient | Age, years | Sex | MM type | Bence Jones | ECOG | Prognostic Index (ISS-R) | Treatment when taking the sample | Situation |
| --- | --- | --- | --- | --- | --- | --- | --- | --- |
| 1 | 62 | F | IgG λ | No | 0 | 3 | KyCyDex | Partial response |
| 2 | 66 | M | BJ κ | Yes | 0 | 2 | Untreated | Progression |
| 3 | 82 | M | IgG κ | No | 0 | 3 | Untreated | Progression |
| 4 | 69 | F | IgG κ | No | 0 | 1 | Untreated | Progression |
| 5 | 68 | F | IgG κ | No | 1 | 3 | Kd | Very good Partial response |
| 6 | 68 | M | IgG λ | No | 3 | Not calculable | Untreated | Progression |
| 7 | 79 | M | IgG κ | No | 1 | Not calculable | CLARIDEX | Partial response |
| 8 | 36 | F | IgG λ | No | 0 | 1 | Rd | Complete response (MRD+) |
| 9 | 77 | F | IgG κ | No | 0 | Not calculable | Rd | Partial response |
| 10 | 59 | M | IgG κ | No | 0 | 3 | VRD | Partial response |
| 11 | 73 | F | IgG λ | No | 0 | 1 | KyCyDex | Partial response |

**Supplemental Table S1. Clinical characteristics of the analyzed multiple myeloma patients.** Samples from multiple myeloma patients were used for CAR transduction experiments. F: female; M: male; BJ: Bence Jones; KyCyDex: carfilzomib, cyclophosphamide, and dexamethasone; Kd: carfilzomib and dexamethasone; CLARIDEX; clarithromycin, lenalidomide and dexamethasone; Rd: lenalidomide and dexamethasone; VRD: bortezomib, lenalidomide, and dexamethasone; MRD: minimal residual disease.

**Table S2**

| Antigen | Clone | Fluorochrome | Source | Catalog number |
| --- | --- | --- | --- | --- |
| CD3 | UCHT1 | PE/Cy7 | Biolegend | 351304 |
| CD7 | CD7-6B7 | FITC | Biolegend | 343104 |
| CD16 | 3G8 | APC/Cy7 | BD Pharmingen | 557758 |
| CD25 | BC96 | FITC | Biolegend | 302604 |
| CD45 | HI30 | FITC | Biolegend | 304006 |
| CD56 (NCAM) | HCD56 | APC | Biolegend | 318310 |
| CD69 | FN50 | PE | Biolegend | 310906 |
| CD178 (FasL) | NOK-1 | PE | Biolegend | 306407 |
| CD253 (TRAIL) | RIK-2 | PE | Biolegend | 308206 |
| DNAM-1 (CD226) | TX25 | FITC | Biolegend | 337104 |
| CD94 | DX22 | FITC | Biolegend | 305504 |
| NKG2A (CD159a) | #131411 | PE | R&D Systems | FAB1059P |
| NKG2D (CD314) | 1D11 | PE | Biolegend | 320806 |
| NKp30 | AF29-4D12 | PE | Miltenyi | 130-092-483 |
| NKp44 | Z231 | PE | IOtest | PNIM3710 |
| NKp46 | 9E2 | PE | Biolegend | 331908 |
| NKp80 | 5D12 | PE | Biolegend | 346706 |
| CD45RA | HI100 | FITC | Biolegend | 304106 |
| DAPI |  |  | Sigma Aldrich | D9542 |
| MICA | #159227 | PE | R&D Systems | FAB1300P |
| MICA/B | 6D4 | PE | Biolegend | FAB131001P |
| ULBP-1 | #170818 | PE | R&D Systems | IC1380P |
| ULBP-2/5/6 | #165903 | APC | R&D Systems | FAB1298A |
| ULBP-3 | #166510 | PE | R&D Systems | FAB1517P |

**Supplemental Table S2. Antibodies used in flow cytometry studies.**FITC, fluorescein isothiocyanate; PE, phycoerythrin; PE/Cy7, tandem comprising phycoerythrin and cyanine 7; APC, allophycocyanin; APC/Cy7, tandem comprising allophycocyanin and cyanine 7.

**Supplementary Video Legend**

MM cells from U-266 cell line (green fluorescent cells) were seeded forming a monolayer on a six-channel polymer-treated flow chamber. Then a constant flux of either CAR-NKAE cells (red fluorescent cells, left) or memory CAR-T cells (red fluorescent cells, right) from the same MM patient was established over 20 min. Subsequently, four-hour observation of CAR-NKAE cell and memory CAR-T cell activity was performed. Supplemental video can be found in a public repository: “Time-lapse microscopy of CAR-NKAE cells and memory CAR-T cells from multiple myeloma patients”, Mendeley Data, V1, doi: 10.17632/m79ygj3rwr.1
